# Long-term effects of delayed graft function duration on function and survival of deceased donor kidney transplants

**DOI:** 10.1590/2175-8239-JBN-2018-0065

**Published:** 2018-10-04

**Authors:** Mateus Swarovsky Helfer, Jeferson de Castro Pompeo, Otávio Roberto Silva Costa, Alessandra Rosa Vicari, Adriana Reginato Ribeiro, Roberto Ceratti Manfro

**Affiliations:** 1 Universidade Federal do Rio Grande do Sul Porto AlegreRS Brasil Universidade Federal do Rio Grande do Sul, Porto Alegre, RS, Brasil.; 2 Hospital de Clínicas de Porto Alegre Nefrologia Porto AlegreRS Brasil Hospital de Clínicas de Porto Alegre, Nefrologia, Porto Alegre, RS, Brasil.

**Keywords:** Kidney Transplantation, Delayed Graft Function, Graft Survival, Survival Analysis, Graft Rejection

## Abstract

**Introduction:**

Delayed graft function (DGF) is a frequent complication after deceased donor kidney transplantation with an impact on the prognosis of the transplant. Despite this, long-term impact of DGF on graft function after deceased donor kidney transplantation has not been properly evaluated.

**Objective:**

The main objective of this study was to evaluate risk factors for DGF and the impact of its occurrence and length on graft survival and function.

**Methods:**

A retrospective cohort study was performed in 517 kidney transplant recipients who received a deceased donor organ between January 2008 and December 2013.

**Results:**

The incidence of DGF was 69.3% and it was independently associated with donor's final serum creatinine and age, cold ischemia time, use of antibody induction therapy and recipient's diabetes mellitus. The occurrence of DGF was also associated with a higher incidence of Banff ≥ 1A grade acute rejection (P = 0.017), lower graft function up to six years after transplantation and lower death-censored graft survival at 1 and 5 years (P < 0.05). DGF period longer than 14 days was associated with higher incidence of death-censored graft loss (P = 0.038) and poorer graft function (P < 0.001). No differences were found in patient survival.

**Conclusions:**

The occurrence of DGF has a long-lasting detrimental impact on graft function and survival and this impact is even more pronounced when DGF lasts longer than two weeks.

## INTRODUCTION

Delayed graft function (DGF) is a frequent complication after deceased donor kidney transplantation with an impact on the prognosis of the transplant.^1^ DGF has many definitions and currently the most commonly employed is the need for dialysis within the first week after transplantation.^2^ Its overall incidence, according to the Organ Procurement and Transplantation Network/Scientific Registry of Transplant Recipients 2012 Annual Data Report, is stable around 24% in the United States of America.^3^ However, much higher incidence rates have been observed in many countries, particularly in Brazil, for reasons that are not entirely clear but that are probably related to suboptimal donor maintenance.^4^

The consequences of the high DGF incidence are striking in terms of costs, morbidity, and perhaps mortality.^5^ The delay in recovering renal graft function results in prolonged hospitalization and therefore higher cost to health care systems. Furthermore, DGF is associated with a higher incidence of acute rejection, worse graft function and poorer graft survival. Additionally, higher mortality has been reported in patients with DGF.^5^

Even though the impact of this condition has been extensively reported, the consequences of DGF length on graft survival and function are less certain. It is conceivable that prolonged duration of DGF may be associated with inferior graft outcomes. The present study was undertaken to evaluate the risk factors for DGF and the impact of its duration in long-term kidney graft survival and function.

## MATERIALS AND METHODS

We performed a retrospective cohort study that included 517 kidney transplant recipients who received a deceased donor organ between January 2008 and December 2013 at our institution. Twenty-eight patients were withdrawn from study analysis since the diagnostic criteria for DGF could not be evaluated due to primary non-function, early deaths, and early graft losses. DGF was defined by the need for dialysis in the first week after transplantation; all patients were followed for at least one year and up to six years. Risk factors for DGF were evaluated along with recipient demographic data such as age, gender, race, time on renal replacement therapy, primary kidney disease, HLA mismatches, presence of donor-specific antibodies, panel-reactive antibodies, and immunosuppressive regimen including the use of antibody induction therapy. Donor-related variables included demographic data, final serum creatinine, cause of death, history of hypertension, and being classified as expanded criteria donors (ECD).^6^ Graft- and transplant surgery-related factors were: cold ischemia time, pre-implantation biopsy (mostly indicated in donors with initial and pre-retrieval serum creatinine higher than 1.5 mg/dL and 4.0 mg/dL, respectively, diabetic donors and in donors older than 65 years old) and organs coming from another Brazilian state and transplanted in our center as per the Brazilian regulations for organ allocation ("out-of-state" organs). Standard multi-organ retrieval technique and kidney transplant anesthesia protocols were used. All kidneys underwent static preservation. Cold ischemia time was measured from the organ cooling within the donor up to being withdrawn from preservation solution, and warm ischemia time was measured from this point up to vascular clamps release. Transplant surgeries were performed according to routine well-established surgical techniques by experienced transplant surgeons.

Duration of DGF was measured in days and the last DGF day was considered the one in which the last dialysis treatment was undertaken. The outcomes evaluated were: (a) incidence of DGF; (b) incidence of acute rejection evaluated throughout the follow-up period; (c) estimation of graft glomerular filtration rate (eGFR) by the MDRD equation according to the presence of DGF and its duration; (d) Kaplan-Meier analysis of patient and graft survival according to the presence or absence of DGF and DGF length.

Statistical analyzes included normality evaluations of the data performed by Shapiro-Wilk and Kolmogorov-Smirnov tests. Turkey's test was used for the analysis of DGF into quartiles according to its duration. The variables were subjected to univariate analysis and those that reached a P level ≤ 0.20 were included in a multivariable analysis by Poisson regression in order to independently evaluate risks factors.

All data analyses were performed with the IBM SPSS Statistics program version 20 and a P value < 0.05 was required for statistical significance.

The study was approved by the institution's ethics and research committee.

## RESULTS

### Demographic data, risk factors, and incidence of DGF

Demographic data is shown in Table 1. Patients were predominantly middle-aged white males, unsensitized that received a first graft. One third of the recipients were grafted with kidneys from expanded criteria donors (ECD).^6^

**Table 1:** Demographic data of the recipients, donors, and transplant variables

Recipients variables	All patients	With DGF	Without DGF	P
	(N = 517*)	(N = 339)	(N = 150)	
Age (years, mean ± SD)	49.2±0.6	49.3±0.7	49.0±1.0	.819
Race (% white)	84.71	88.20	92.66	.325
Gender (% male)	54.93	58.40	57.33	.843
Time on RRT (months, mean±SD)	52.7±3.3	51.8±2.2	54.6±9.6	.699
PRA Class I (%)	17.1±1.3	16.2±1.5	19.2±2.42	.267
PRA Class II (%)	15.0±1.15	14.5±1.38	16.1±2.06	.500
Presence of DSAs** (% with)	16.24	16.81	18.00	.895
HLA (ABDR) mismatches (mean±SD)	3.32±0.05	3.39±0.06	3.17±0.08	.037
Induction Therapy (%)	84.13	92.92	80.0	.000
Previous transplantation (%)	8.89	8.55	11.33	.400
Primary kidney disease				
Hypertension (%)	24.56	23.30	29.33	.259
Diabetes mellitus (%)	21.08	23.01	18.0	.234
Polycystic kidney disease (%)	12.77	11.50	16.0	.188
Chronic glomerulonephritis (%)	7.74	8.85	6.67	.478
Obstructive uropathy (%)	4.45	4.13	4.67	.811
Systemic lupus erythematosus	1.16	1.18	1.33	.888
Others (%)	8.32	7.96	6.00	.715
Unknown (%)	19.92	20.06	18.0	.623
Donor variables				
Age (years, mean ± SD)	43.7 ± 0.77	45.7±0.87	39.2±1.52	.000
Gender (% male)	52.41	56.93	52.00	.323
ECD (% yes)	29.01	33.04	25.33	.105
Serum creatinine*** (mg/dL)	1.60 ± 0.05	1.75±0.07	1.26±0.06	.000
History of hypertension (% yes)	25.91	30.68	20.00	.003
Causes of death				
Cerebrovascular disease (%)	56.03	57.57	50.00	.234
Trauma (%)	34.63	34.12	39.33	.251
Anoxia/drowning (%)	4.28	3.56	5.33	.417
Others (%)	5.06	4.75	5.33	.214
Transplant related variables				
Cold ischemia time (hours, mean±SD)	21.9 ± 0.25	22.49±0.31	20.6±0.41	.001
Outstate organ (%)	18.57	23.30	10.67	.002
Pre-implant biopsy performed (%)	39.65	47.20	30.0	.000

* 28 patients were excluded, see text;

** donor-specific antibodies

*** Last serum creatinine before organ recovery

Risk factors were classified as donor-related, recipient-related, and graft-related. They were analyzed by univariate and multivariate methods (Table 2). Donor's age, final serum creatinine, and history of hypertension were significant risk factors in the univariate analysis (P < 0.05). Among the recipient-related variables, only the use of antibody induction therapy (P = 0.002) and the number of HLA ABDR mismatches (P = 0.034) were significant risk factors. The graft-related variables cold ischemia time, out-of-state kidneys, and having had a pre-implantation biopsy for an evaluation of graft adequacy for transplantation, were significant risk factors for DGF in the univariate analysis (P < 0.001).The variable out-of-state kidneys presented significant co-linearity with cold-ischemia time (P < 0.01) and with pre-implantation biopsy (P < 0.01), and for this reason was not included in the multivariate analysis model. The variables that remained significant in the multivariate analysis model were: donor final serum creatinine (P = 0.012), donor age (P = 0.003), cold ischemia time (P = 0.018), and use of antibody induction therapy (P = 0.004). ECD definition components (donor age, creatinine, history of hypertension, and death by cerebrovascular disease) were entered individually in the multivariate analysis.

**Table 2:** Analysis of risk factors for delayed graft function

Variable	RR (95% CI)	P
Univariate Analysis		
Donor related		
Age (years)	1.007 (1.003 - 1.011)	.000
Gender	1.061 (0.941 - 1.196)	.335
ECD (UNOS)	1.111 (0.984 - 1.255)	.090
Final serum creatinine (mg/dL)	1.116 (1.072 - 1.161)	.000
Hypertension	1.273 (1.091 - 1.485)	.002
Cerebrovascular death (%)	0.903 (0.780 - 1.046)	.175
Trauma as cause of death	0.928 (0.817 - 1.054)	.252
Anoxia/drowning as cause of death	1.090 (0.901 - 1.316)	.379
Other cause of death	1.092 (0.957 - 1.245)	.192
Recipient-related variables		
Age (years)	1.000 (0.996 - 1.005)	.855
Gender	1.015 (0.901 - 1.144)	.805
Hypertension	0.919 (0.780 - 1.046)	.255
Diabetes mellitus	1.092 (0.957 - 1.245)	.192
Polycystic kidney	0.878 (0.717 - 1.076)	.210
Chronic glomerulonephritis	1.089 (0.901 - 1.316)	.379
Systemic lupus erythematosus	0.960 (0.544 - 1.696)	.960
Obstructive uropathy	0.982 (0.733 - 1.314)	.902
Time on dialysis (months)	1.000 (0.999 - 1.001 )	.727
Presence of DSAs (%)	0.983 (0.832 - 1.161)	.843
PRA class I (%)	0.999 (0.996 - 1.001)	.237
PRA class II (%)	0.999 (0.997 - 1.002)	.412
HLA ABDR mismatches	1.059 (1.004 - 1.117)	.034
Antibody induction therapy (%)	1.631 (1.204 - 2.210)	.002
Previous transplantation (%)	0.898 (0.714 - 1.129)	.357
Graft-related variables		
Cold ischemia time (hours)	1.019 (1.008 - 1.029)	.000
Outstate kidney (%)	1.245 (1.108 - 1.400)	.000
Need for preimplantation biopsy (%)	1.305 (1.140 - 1.494)	.000
Multivariate Analysis		
Cold ischemia time	1.018 (1.002 - 1.203)	.018
Donor age	1.007 (1.003 - 1.011)	.000
Donor final serum creatinine	1.099 (1.054 - 1.145)	.000
Antibody induction	1.479 (1.101 - 1.988)	.009

*donor-specific antibodies

The most frequent recipient causes of chronic renal failure were hypertensive nephropathy (24.6%), diabetic nephropathy (21.1%), adult polycystic kidney disease (12.8%), and chronic glomerulonephritis (7.7%). Donor's causes of death were predominantly cerebrovascular disease (56.0%) and trauma (34.6%), which were not risk factors for DGF (data not shown).

DGF occurred in 339 patients, reaching an incidence of 69.3%. The incidence was elevated over the six yearly cohorts ranging from 59.8% in the lowest and 74.4% in the highest incidence year. Two hundred and eighty patients (57.3%) would have been considered as having DGF if the condition was defined by the need of more than one dialysis session in the first post-transplant week.

### Impact of DGF and its duration on graft function

**Figure 1:**
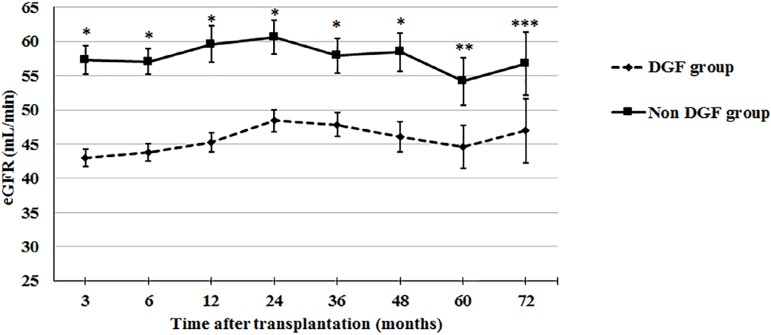
MDRD estimated glomerular filtration up to 72 months after transplantation according to the occurrence of delayed graft function. * P < 0.01; ** P = 0.072; *** P = 0.219

**Figure 2:**
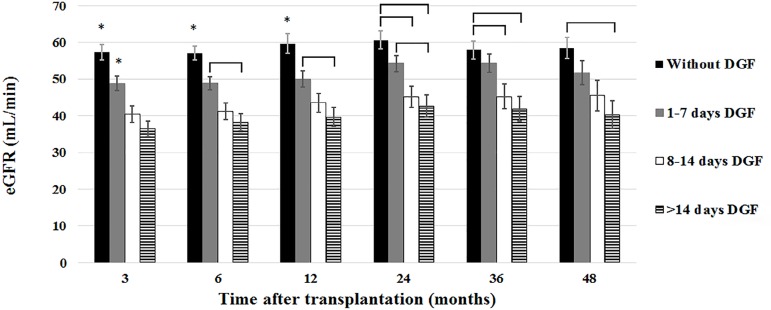
MDRD estimated glomerular filtration rate up to 48 months after transplantation according to delayed graft function duration. * P < 0.05 versus all other groups; brackets indicate P< 0.05

The impact of DGF on graft function is shown in Figure 1, which shows MDRD estimated GFR up to 72 months after transplantation. In the group of patients without DGF, eGFR was significantly higher up to four years after transplantation (P < 0.001) but the difference lost significance at 60 months (P = 0.072) and at 72 months (P = 0.219). In order to analyze the impact of DGF duration on kidney graft function, patients were classified into four groups: (a) without DGF (N = 150), (b) with DGF duration between 1-7 days (N = 154), (c) with DGF duration between 8-14 days (N =81), and (d) with DGF duration longer than 14 days (N = 104). The effects of DGF duration on eGFR are shown in Figure 2. Up to four years after transplantation, a stepwise drop in eGFR was observed as DGF lasted longer. The differences lost statistical significance at 60 and 72 months due to graft losses that occurred predominantly in the group of patients with poorer eGFR. Even a short DGF period presented a detrimental effect on eGFR that lasted throughout the period of observation. The group of patients with longer DGF duration presented the poorest renal function and the group with a DGF lasting 8 to 14 days presented intermediate eGFR values.

### Impact of DGF and its duration on patients and grafts survival

**Figure 3:**
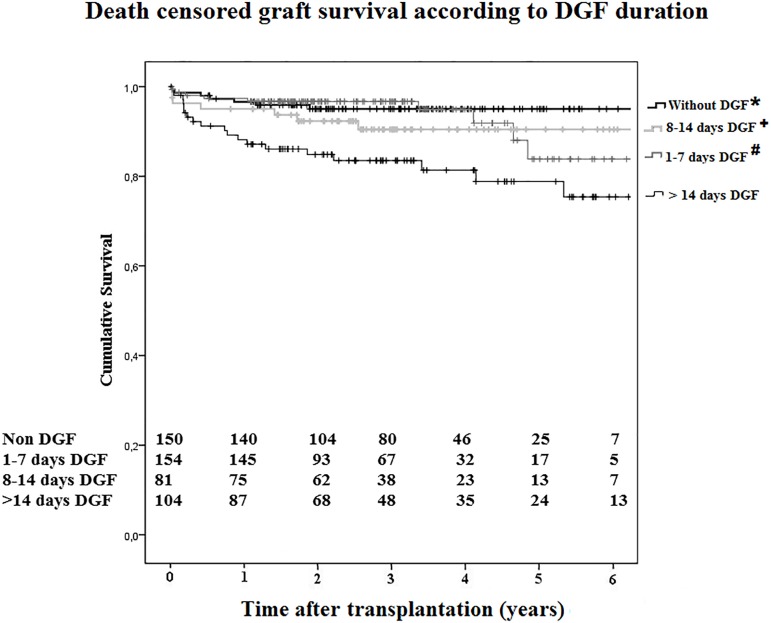
Death censored graft survival according the occurrence and duration of DGF. * P = 0.001 versus group >14 days DGF; # P = 0.006 versus group >14 days DGF; + P = 0.076 versus group >14 days DGF

Overall, prior to any exclusion, patients and uncensored grafts survivals at one and five years after transplantation were 95.6% and 86.5%, and 84.0% and 69.1%, respectively. At one year after transplantation, patient survival in the DGF and non-DGF groups were 96.5% and 96.0%, respectively and remained essentially identical throughout the follow-up (log rank P = 0.601). However, a statistically significant difference was found in death-censored graft survival (P = 0.038). At one year after transplantation, graft survival was 94.0% (DGF group) and 96.6% (non-DGF group), and at five-year, survival were 84.6% and 95.0%, respectively (P = 0.038). Sixty-five graft losses occurred in the follow-up. The main causes of graft loss were vascular thrombosis in 26 cases (40%), rejection in 14 cases (21.5%), and chronic allograft failure in 13 cases (20%). DGF duration did not impact on patient survival up to 6 years after transplantation; however, it exerted a significant impact on graft survival. The group of patients with DGF longer than two weeks presented a significant lower graft survival (Figure 3).

### Impact of acute rejection and DGF on graft function and survival

**Figure 4:**
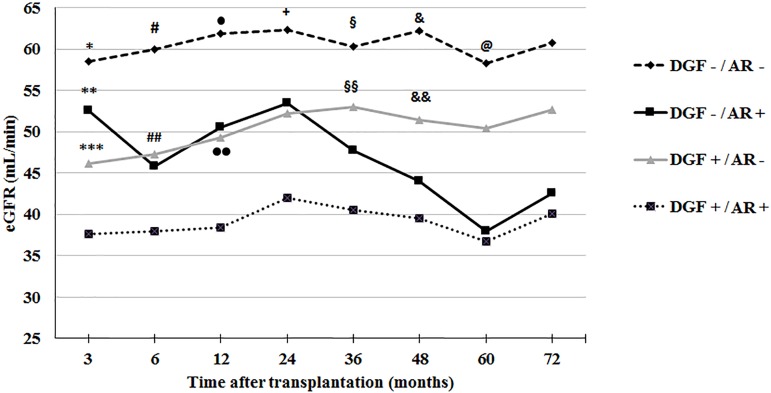
MDRD estimated glomerular filtration rate up to 72 months after transplantation according to the occurrence of delayed graft function and acute rejection evaluated throughout the follow-up period AR = acute rejection; DGF = delayed graft function * P < 0.01 versus DGF+/AR- and DGF+/AR+ ; ** P =0.011 versus DGF+/AR+; *** P = 0.009 versus DGF+/AR+; # P <0.05 versus all other groups; ## P = 0.002 versus DGF+/AR+; • P <0.01 versus DGF+/AR+; •• P=0.003 versus DGF+/AR+; + P < 0.05 versus DGF-/AR+ and DGF+/AR+; § P < 0.01 versus DGF+/AR+; §§ P = 0.003 versus DGF+/AR+; & P <0.01 versus DGF+/AR+; && P = 0.019 versus DGF+/AR+; @ P = 0.006 versus DGF+/AR+

In the first year after transplantation, the incidence of biopsy confirmed (Banff ≥ 1A) that acute rejection was 24.5% in the DGF group and 14.7% in the group of patients without DGF (P = 0.017). Acute rejection superimposed on DGF led to significant lower eGFR and death-censored graft survival. As shown in Figure 4 the group of patients without either DGF or acute rejection presented higher eGFR throughout the observation period and the group with both conditions had lower eGFR. Acute rejection and DGF presented similar impacts on eGFR up to three years after grafting. After this period, patients with previous acute rejection presented a more significant decrement of eGFR.

At six years after transplantation, death-censored graft survivals were: 95.5% in the group of recipients without either DGF or acute rejection, 93.1% in the group without DGF and with rejection, 89.4% in the group with DGF and without rejection, and 73.9% in the group with both DGF and acute rejection. The group without DGF or rejection presented significantly higher survival than the group of patients with both conditions (P < 0.001). Also, the survival of the group without DGF and with acute rejection was higher than the group with both conditions (P < 0.001).

## DISCUSSION

Delayed graft function is a frequent complication after deceased donor renal transplantation and presents as graft acute renal failure resulting many times in post-transplantation oliguria, need for dialysis, increased allograft immunogenicity with higher risk of acute rejection, and may lead to decreased graft survival.^7^ Its impact in patient survival is less clear and previous reports show either a significant decrease or no impact.^5^^, ^8

Donor-related factors may influence the occurrence of DGF, notably quality of donor intensive care during organ retrieval.^9^ Recipient-related risk factors may be classified in immunologic and non-immunologic. The immunologic risk factors include HLA mismatches, pre-transplant PRA, and blood transfusions.^10^ Reported non-immunologic risk factors are donor age, cold ischemia time, gender mismatch, gender, weight, ethnicity, and medical status.^11^

The incidence of DGF after deceased donor kidney transplantation presents a wide variation. The current reported average incidence in US is around 30 and 22% for kidney grafts from extended and standard-criteria donors, respectively.^3^ Apparently, this large variability results mainly from differences in the rates reported by transplantation registries, whether heart-beating or non-heart-beating donors were included, as well as the ambiguity in DGF definition.^2^ Furthermore, local factors seem to impact in the incidence. In Brazil, an incidence of 55.6% was reported in a retrospective multicenter study.^12^More recently, single center studies reported even higher incidences.^3^^, ^7^, ^13

In a recent North-American registry study, outcomes of 29.598 mate kidney transplants from the same donor were evaluated, where only one transplant underwent DGF. The authors found that the risk of graft loss associated with DGF in the first year after transplantation was 5.35 times higher and remained between 16 and 30% higher after the first year.^14^

In the present work, the incidence of DGF was significantly higher than the mean incidence in the US, and similar with previous Brazilian reports.^3^^, ^13^, ^15 The retrospective nature of the present work does not allow the identification of the causes for such finding, however one can speculate that this outcome is mostly related to donor care, procurement and retrieval of organs, and donor characteristics.^16^Non heart-beating donors are currently not allowed in Brazil, however a high percentage of expanded-criteria donor organs use is common in most Brazilian transplant centers. In addition, prolonged cold ischemia times and out-of-state organs might contribute to the elevated incidence of DGF found in this country. According to the regulations of the Brazilian transplant system, retrieved organs that are not accepted for transplantation in the state of retrieval must be offered nationally. Depending on the accepting states, organs are allocated to patients in higher need or better logistics. For these reasons, many of these organs came from expanded criteria and not optimally maintained donors. In fact, in the present cohort, out-of-state kidneys underwent more frequent pre-implantation biopsies and were transplanted after longer cold ischemia times.

Renal grafts from expanded criteria donors have also been associated with a higher DGF incidence compared to grafts from standard criteria donors 3. In the present work, donor's age, final serum creatinine, and history of hypertension had a strong association with DGF in the univariate analysis. Extended cold ischemia time has also been described as an independent risk factor for DGF. Data from the US Renal Data System Registry indicated a 23% increase in risk for every 6 hours of increase in the cold ischemia. In our report, the main graft-related risk factors associated with DGF were cold ischemia time, out-of-state kidneys, and the need for a pre-implantation biopsy. In the DGF group, 23% of the grafts came from other states while only 10% of the grafts came from other states in the group that did not undergo DGF. Organs made available by another state are usually from ECD, driving the need for a pre-implantation biopsy and are submitted to longer cold ischemia times.

In the present work, we examined, in a setting of high DGF incidence, the risk factors and prognostic significance to verify if poorer DGF-related outcomes would sustain. As previously reported by Brazilian studies, an elevated incidence of DGF was found.^4^^, ^12^, ^13^, ^15 In the multivariate analysis model, the variables that remained statistically significant were donor final creatinine, donor age, cold ischemia time, and use of antibody induction therapy. As expected, patients who received kidneys with acute renal failure were more likely to undergo DGF, as also observed in other studies. 4^,^^17^ Due to its retrospective design, the reasons why antibody induction therapy emerged as an independent risk factor could not be uncovered in this study. We believe that antibody induction was preferentially indicated for transplants with less favorable profiles of donors and/or recipients, such as prolonged cold ischemia time, donor with acute renal failure, and broadly sensitized recipients.

Tedesco-Silva and collaborators showed that machine perfusion of kidneys grafts significantly decreased the incidence of DGF from 61 to 45% compared with static preservation in a recent multicenter Brazilian study.^18^ Also in that study, machine perfused organs presented better function at 1 and 12 months after transplantation. In another prospective Brazilian cohort by Matos et al, static preservation was shown to increase the risk of DGF by 54% in comparison with machine perfusion. Besides, DGF length was significantly shortened by more than 50% in the machine perfused arm of the study.^19^

Interestingly, the impact of DGF length on graft outcomes has not been frequently reported.^5^^,^^17^^, ^20 In the present study, we found that DGF duration has no impact on patient survival but an evident detrimental effect of DGF lasting longer than 14 days on graft survival was observed. In support, Sandes-Freitas and collaborators recently reported significantly lower short-term graft survivals in kidney transplant recipients with prolonged DGF (> 15 days).^13^ In long-term studies, Yokoyama et al. reported higher frequency of graft failure at 5 years after transplantation in the group of patients with DGF longer than 8 days.^21^ Also, Fernández-Juarez and collaborators described lower graft survival up to 6 years after transplantation in the group of patients with DGF longer than two weeks.^22^ However, in the latter report, when primary non-function grafts were excluded, the differences in graft survival disappeared. In the present study, patients who could not meet the diagnostic criteria for DGF, as well those with primary non-function, were excluded from the analysis.

Graft recovery of function is another important DGF related concern. Lee and collaborators evaluated the impact of DFG recovery on graft function and found that those kidneys with complete recovery have similar survival to the ones that did not have DGF. Contrariwise, those with uncomplete recovery presented lower survival and lower GFR at five years after transplantation. 23

In a meta-analysis study, Yarlagadda and collaborators showed that patients with DGF present lower graft function compared with those without DGF at 3.5 years after transplantation.^7^ Also, Jayaram et al. reported that patients with DGF that required more than one dialysis treatment displayed significant lower renal function after graft recovery.^24^ A significant detrimental impact of DGF length in short-term graft function has also been shown by Sandes-Freitas et al., who reported a significantly worst graft function in patients with DGF longer than 15 days.^13^ Previously, Renkens et al. reported that DGF longer than 30 days in recipients of non-heart beating donor kidneys presented inferior function at three months after transplantation.^20^ In the present work, we also found reductions in eGFR in the group of patients that underwent DGF. Interestingly, eGFR reductions occurred in a stepwise fashion with DGF length, were sustained over the period of six years, and were significantly lower in comparison with the groups of patients without DGF and with the group with shorter DGF duration. It is conceivable that kidneys of DGF patients who required only one-time or a short time of dialysis had suffered less severe ischemia and reperfusion injuries as compared with grafts from patients who require further dialysis treatments. The severity of ischemia and reperfusion injuries could have determined the duration of dialysis requirement and possibly led to maladaptive repair of parenchymal cells leading to fibrosis and inferior long-term clinical outcomes, as demonstrated by the present study.^25^

DGF has been related to a higher incidence of acute rejection in kidney transplant recipients.^7^^,^^24^^,^^26^ A number of mechanisms have been proposed to explain this finding. They include the augmented immunity elicited by ischemia and reperfusion injuries leading to an inflamed environment, with increased release of inflammatory cytokines and MHC class I and II molecules expression on graft cell surfaces, thereby increasing direct and indirect recognition by the host immune system.^27^ In our study, the incidence of biopsy-confirmed acute rejection was higher in the group of patients that underwent DGF. Miglinas et al. also reported higher incidence of biopsy-proven acute rejection episodes in patients with DGF.^10^ However, these findings must be viewed with caution. Patients with DGF are more frequently submitted to surveillance biopsies and are thus more prone to have inflammatory reactions in the uncovered graft. Furthermore, grafts with immediate function may present an elevated incidence of sub-clinical acute rejection, only revealed by early protocol biopsies that are not routinely performed. Also, longer cold ischemia times seems not to be related to a higher incidence of rejection.^28^ In the present study DGF without acute rejection was associated with lower graft survival and the occurrence of acute rejection led to the worst graft survival. Troppman et al. have suggested that DGF without rejection has no impact on long-term graft survival.^29^ Other studies have suggested that graft survival in patients with or without DGF is similar unless acute rejection occurs, in which case a significant worsening of graft survival is observed.^30^^,^^31^ However, there are also reports in which DGF and acute rejection were found to be independent risk factors for allograft failure.^32^^,^^33^

In our study, patient survival was not influenced by the occurrence or duration of DGF up to six years after transplantation. The relationship between DGF and mortality has been evaluated in many studies. Yarlagadda and collaborators performed a systematic review and meta-analysis, including twelve studies, evaluating the impact of DGF on mortality of kidney transplant recipients and found no association between DGF and patient survival up to five years after transplantation.^7^

The present study has limitations including being single center and its retrospective design that prevented a better assessment of a number of aspects, but in particular, the quality of donor care. However, we believe that the data brought out significant findings regarding the impact of DGF length in graft survival and function.

In conclusion, in a setting of high DGF incidence, prolonged DGF is associated with inferior graft survival and function. Acute rejection is more frequent in patients with DGF and its occurrence further aggravates survival and function. The reasons for the high DGF incidence could not be identified in the present study. We speculate that donor maintenance-related factors might be involved in such finding and that better donor management protocols, care, and organ retrieval might help improve this outcome.
